# Design and evaluation of sound-based electronic football soccer training system for visually impaired athletes

**DOI:** 10.1186/s12938-019-0695-5

**Published:** 2019-06-24

**Authors:** Francisco Yandun, Fernando A. Auat Cheein, Daniela Lorca, Omar Acevedo, Cecilia Auat Cheein

**Affiliations:** 1 Department of Electronic Engineering, Universidad Técnica Federico Santa María, Av. España 1680, Valparaiso, Chile; 2 Facultad de Arquitectura, Universiad de Valparaíso, Blanco 951, Valparaiso, Chile; 3 Facultad de Ciencias Médicas, Universidad Nacional de Santiago del Estero, Santiago del Estero, Argentina

**Keywords:** Echo-localization, Visually impaired people, Sports rehabilitation

## Abstract

**Background:**

Several countries encourage the practice of football for rehabilitation and social inclusion purposes. For visually impaired people, football is purely sound-based, where the ball and the players are constantly emitting sounds for localization purposes in the field. However, the task of shooting the ball requires of a non-visually impaired extra person, behind the goal (known as *caller*), whom is punching the four corner of such goal to help the athletes. The presence of the *caller* restricts the self-sufficiency of the players. This work addresses such problem, by presenting a goal for visually impaired players with the aim of enhancing their self-sufficiency.

**Materials and methods:**

The electronic goal is designed with four functionalities for training purposes, by returning sound-based feedback of its position and the places where the ball has impacted. The system is validated with seven volunteers from Chilean Football Soccer National Team. A questionnaire was answered by the players before and after the tests to statically validate the proposed device.

**Results:**

The presented system is portable and designed following a modular criterion suitable for visually impaired people self-assembling. From a test of 350 shootings, the electronic goal showed to enhance the shooting assertiveness from 82 to 92%, and the accuracy from 20 to 56% compared to the traditional *caller*.

**Conclusions:**

The electronic goal showed to enhance the self-sufficiency of athletes, by improving their assertiveness in shooting training. Nevertheless, and according to the responses to the questionnaires, the system needs improvements in its portability and handling.

## Background

Sport is a powerful, low-cost mean to foster greater inclusion and well-being for persons with disabilities [1]. Tackling social inclusion and cohesion through sports can enhance the social bounds of a community, increasing diversity, with the possibility of a higher participation [1, 2].

As stated in [1, 3, 4], there is a significant evidence that sports contribute to social inclusion and cohesion. In particular, for impaired people sports can also offer evidence of rehabilitation [1] that brings direct health benefits for individuals and the society. In this context and according to the World Health Organization [5], more than 285 million people are estimated to be visually impaired worldwide, where 39 million are blind and the rest with low vision. Thus, this work is focused on sports, specifically football soccer, for visually impaired people.

In football soccer, there have been an increasing number of products and techniques focused in tracking the movements of players in the soccer field, in order to evaluate their performance [6–11]. The latter is not trivial since not only provides information about the motion of a single player but also provides a global view of the entire team, the correlations and the covered field associated with strategies implemented by the coach. To this aim, Inertial Measurement Units (IMUs), Global Positioning System (GPS) antennas and artificial vision systems are generally used [8, 12, 13]. Being technology the focus, technologically assisted football soccer training, specially shooting, considers different scenarios and different positions, each one of them aimed at developing or enhancing one specific skill from the player, as shown in [14–20].

For visually impaired athletes, however, the technological development has been focused on improving their daily tasks (see [7, 12, 21–28], and the references therein) and not in the sport, specifically, football soccer. Many reasons might explain this scenario, being the two most important ones: the fact that many countries still do not have a federated football soccer sport [12, 29, 30], and the fact that when compared with classical football soccer, visually impaired teams and matches do not attract enough sponsoring . The latter is not a minor issue, causing that most initiatives finally relies on government policies [1, 3, 4]. In addition, currently there exists a lack of technology specially developed to enhance visually impaired football soccer training.

Due to our close work with the Chilean National Football Soccer Team (CNFST, which is amateur, i.e., non-federated) within our research project related to enhancing daily living tasks of visually impaired people, we were able to detect that athletes were not self-sufficient during training and that they depend on an extra person to practice, specially for shooting the goal. Such situation motivated us to design, build and test an electronic goal that is portable, easy to assemble and de-assembly by visually impaired players and with a sound-based training system inspired in echo-localization techniques. The system was tested and validated with seven athletes from the CNFST.

### Problem statement

According to IBSA (International Blind Sports Federation, [29]), visually impaired athletes fall within the following categories according to their impairment: (type B1) visual acuity poorer than LogMAR 2.60; (type B2) visual acuity ranging from LogMAR 1.50 to 2.60 (inclusive) and/or visual field constricted to a diameter of < 10°; (type B3) visual acuity ranging from LogMAR 1.40 to 1 (inclusive) and/or visual field constricted to a diameter of < 40°. In football, teams are arranged according to such classification (completely formed by B1 players; or mixed with B2- and B3-type players). Thus, sound becomes crucial in every match and each actor (ball, player and goal) should be able to provide of a unique sound. It is to be noted that football players often are young people, who underwent different medical procedures resulting in betterment of general condition [31].

**Fig. 1:**
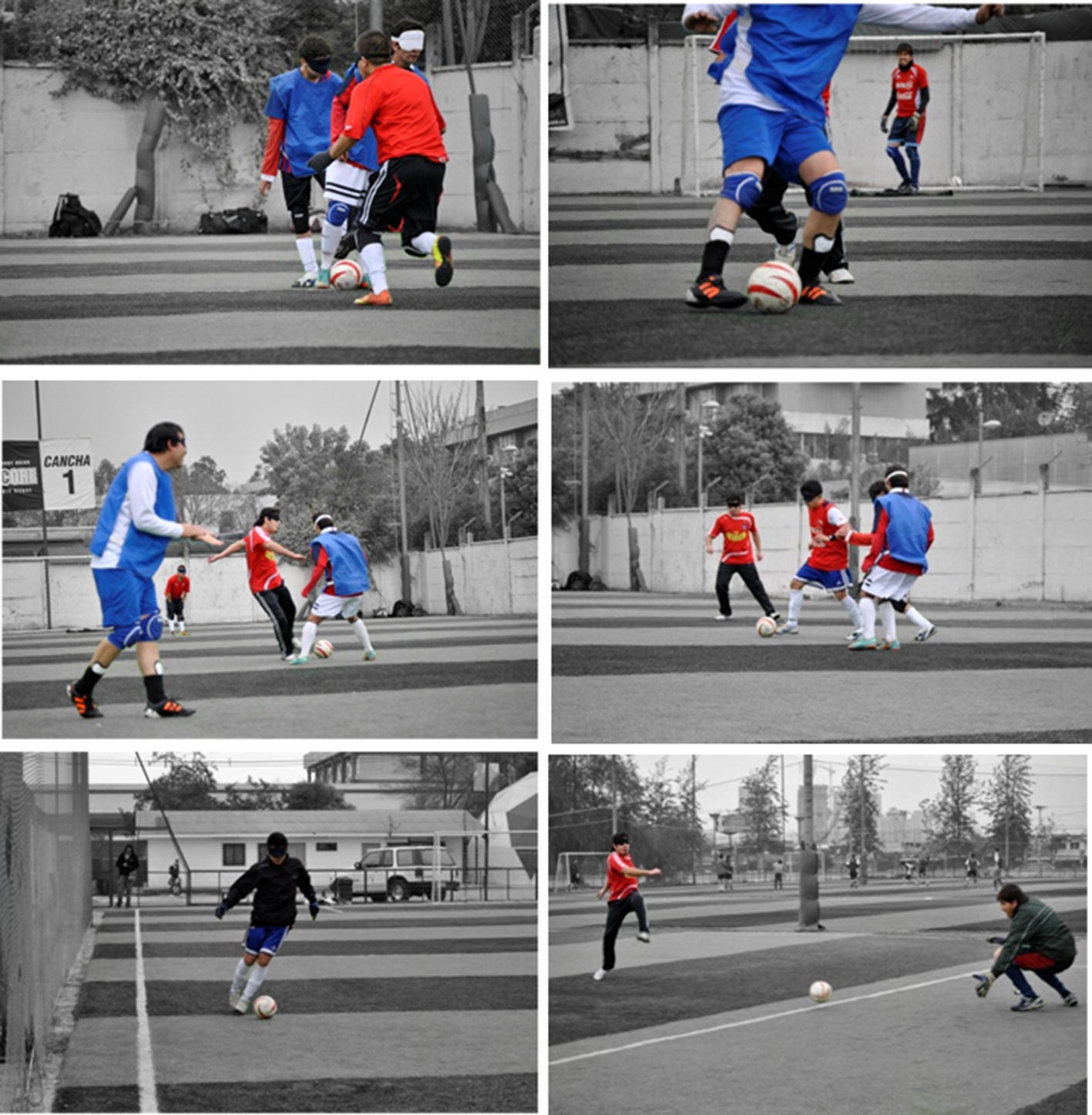
Different snap-shots of the CNFST football soccer practice. It is mandatory for all athletes to wear blindfolds to ensure a fear match

When playing football soccer, the ball is equipped with a sleigh bell inside, thus players can locate the ball within the field by recognizing its sound. Figure 1 shows different snap-shots taken during the CNFST soccer team practice in Santiago, Chile.

**Fig. 2:**
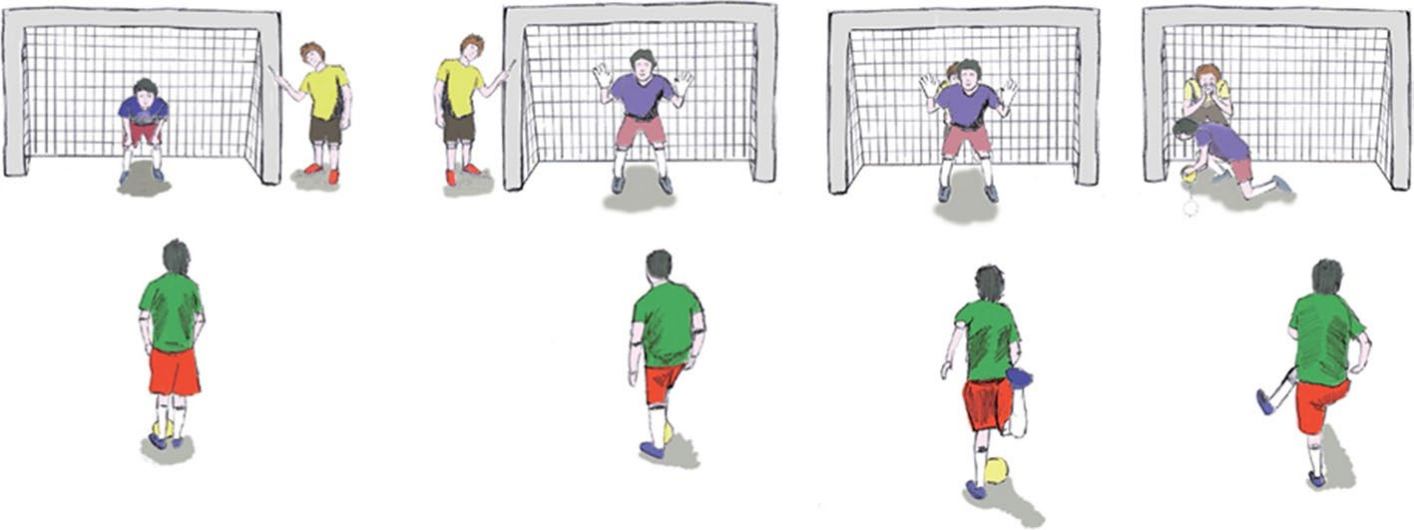
A *caller* before shooting. He or she hits the four corners of the goal and then locates behind the goalkeeper, to indicate him where to move to catch the ball

One of the main issues that athletes have to face is, once located the goalkeeper, they have to locate the goal in the field and to realize its dimensions. Therefore, two extra persons are necessary in the field; they are known as *callers* and their function is to hit the four corners of each goal. The sounds they produce allow the players to interpret the dimensions of the goal and to locate themselves with respect to the goal. The *caller* is usually an assistant (not visually impaired). The *caller* is used both when playing and when practizing shootings (penalties). Figure 2 shows a drawing of the *caller* and their tasks.

For players, the fact of needing an extra person behind the goal hitting the corners constrains their self-sufficiency, since they become dependent on such person. Although during a soccer match the *caller* must be present, during training (whether in team or alone) the presence of the *caller* may not be required. This paper is focused on developing a sound-based electronic goal to enhance the self-sufficiency of blind impaired soccer athletes by neglecting the need of a *caller* during training. To do so, the electronic goal must be portable, designed for the visually impaired for self-assembling and adequate for training. The latter implicitly presents the basic functionalities of the electronic goal, detailed following, but previously agreed upon the athletes:

1. It should be able to emit a sound near each corner of the goal, to allow positioning of the player with respect to it.
2. It should emit a sound according to the place where the ball impacted.
3. It should allow for training shooting sessions, i.e., to emit a sound from a random place from the goal and, according to where the ball impacted, to re-emit the same sound (in case of coincidence) or a different one, thus informing the athlete regarding his success.
4. The goal should allow both indoor and outdoor trainings. Therefore, its electronics and energization system should be properly designed.
5. It should be portable, easy to handle and ease to assemble/de-assemble by visually impaired persons. To this end, a modular design was adopted in this work.
6. It should be robust enough to receive ball impacts without damaging the system.

The following sections are aimed at presenting the sound-based electronic goal developed in this work and its validation with the CNFST.

## Materials and methods

The previous six constraints serve as guidelines for the electronic goal designing process as shown following:

- To solve the first constraint, four speakers at each corner of the metallic goal would have been enough to replace the *caller*. However, such solution would not fulfill requirements 2 and 3.
- A solution based on artificial vision systems to overcome the previous counterparts would have required of calibration and likely the assistance of an extra person to positioning the hardware. The later contradicts requirement 5. Since vision systems are sensitive to lighting conditions, then requirement 4 would be compromised.
- Instead of vision systems, range technology could be used, such as LiDARs and ultrasonic sensors. LiDARs are rather expensive and need of installation and had rather short range of reading; on the other hand, ultrasonic sensors are not only cheap but also need to be installed (see requirement 5). Structured light vision systems, like the Kinect V2, were also considered as a possible solution, but its range is too short compared to the iron goal width.
- Solutions (using LiDARs, ultrasonic sensors or vision systems) must be robust from a construction point of view, since they are likely to receive ball impacts thus causing severe damages to the device (a ball impacting an LiDAR will probably break it since such sensor is not designed to operate under high vibrations or direct impacts. The same applies to vision and ultrasonic sensors).

Therefore, the solution shown in this work is based on the design of a modular and portable electronic goal that can be seen as a sensor network: each module has accelerometers to sense the impact, a speaker to emit a sound and it was built to resist ball impacts. Such design fulfils the six constraints as will be shown in the following sections.

### Goal design

**Fig. 3:**
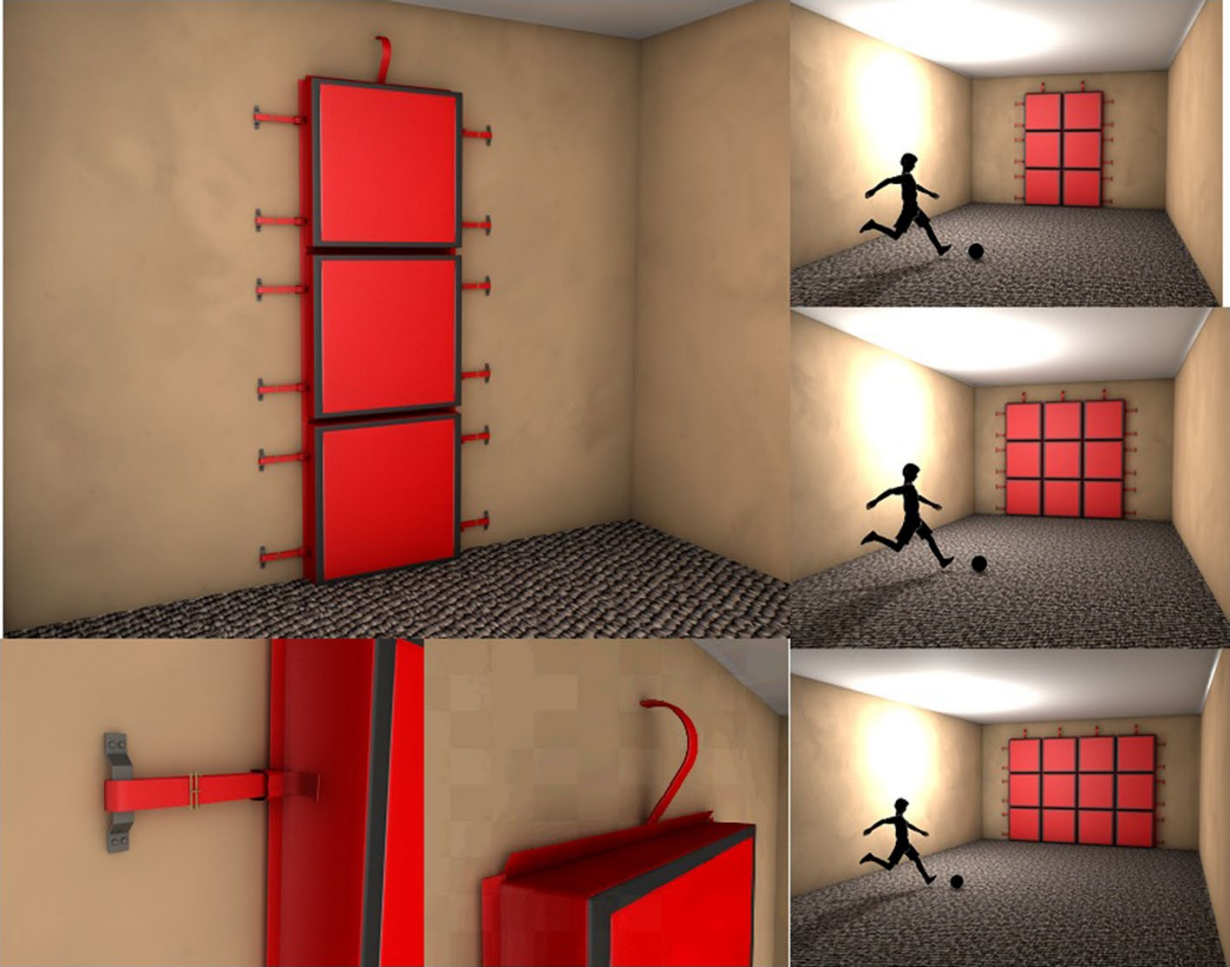
Different drawings of the designed electronic goal. Each column is one module and it is assembled in a unique way with respect to the others

**Fig. 4:**
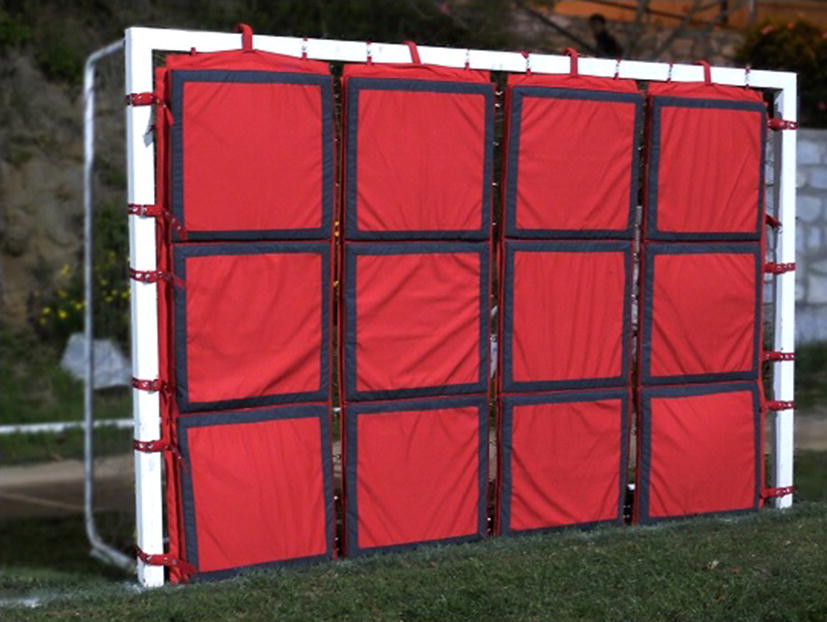
Picture of the electronic goal developed in this work mounted on an iron goal

With the aim of designing a portable (and modular) device that could be located on the actual iron goal (requirements 4–6), a grid-based approach was addressed in this work. Following the results, shoot percentages and information presented in [32] and the goal was divided into a $3\times 4$ grid. Three cells from one column constitute a module to assemble. Therefore, the electronic goal is composed by 4 columns, with 3 cells each. The four columns are assembled in one way only, as will be shown later. Figure 3 shows several drawings of the electronic goal, emphasizing its modularity, given by the fact that the athlete assembles one column at a time and that the goal does not only necessarily need to be located on an actual iron goal, but also in a wall. Figure 4 shows the actual electronic goal built in this work, mounted on a professional iron goal.

The fact of designing a grid of size $3\times 4$ is based on the following:

- Each cell from the grid has an impact sensor and a speaker. The impact sensor detects when the ball hits that cell—or any part of it—and the speaker emits a unique sound associated with such cell.
- Three cells from a column share a micro-controller that processes the impact signals from all sensors and determines which cell has been hit by the ball, thus sending the appropriate sound through the cell’s speaker.
- Addressing the modularity design criterion, each module of the electronic goal is one column with 3 cells. Each column is independent from the others but they physically connect each other in a unique way.
- Further resolution of the grid would have increased its costs and consumed more energy, since each cell has one speaker in it. The $3\times 4$ resolution of the actual grid was a recommendation made by the football soccer trainers based on their experience.

A more detailed description of the electronic perspective of the goal is included in the following section.

### Electronics design

The design of the electronic goal was faced attending three principles: portability, modularity and self-assembly by visually impaired people, requirements 1–3 in “Problem statement” section. As stated in the previous section, following the recommendations given the football soccer trainers, the electronic goal was built according a grid-based system of 12 cells, in 4 columns of 3 cells. Each column assembles the next one in a unique manner since each column has a Braille label that identifies it. Details about the concept design of the electronic goal will be avoided in this manuscript. Instead, we will focus on its electronic design, usability and test results.

**Fig. 5:**
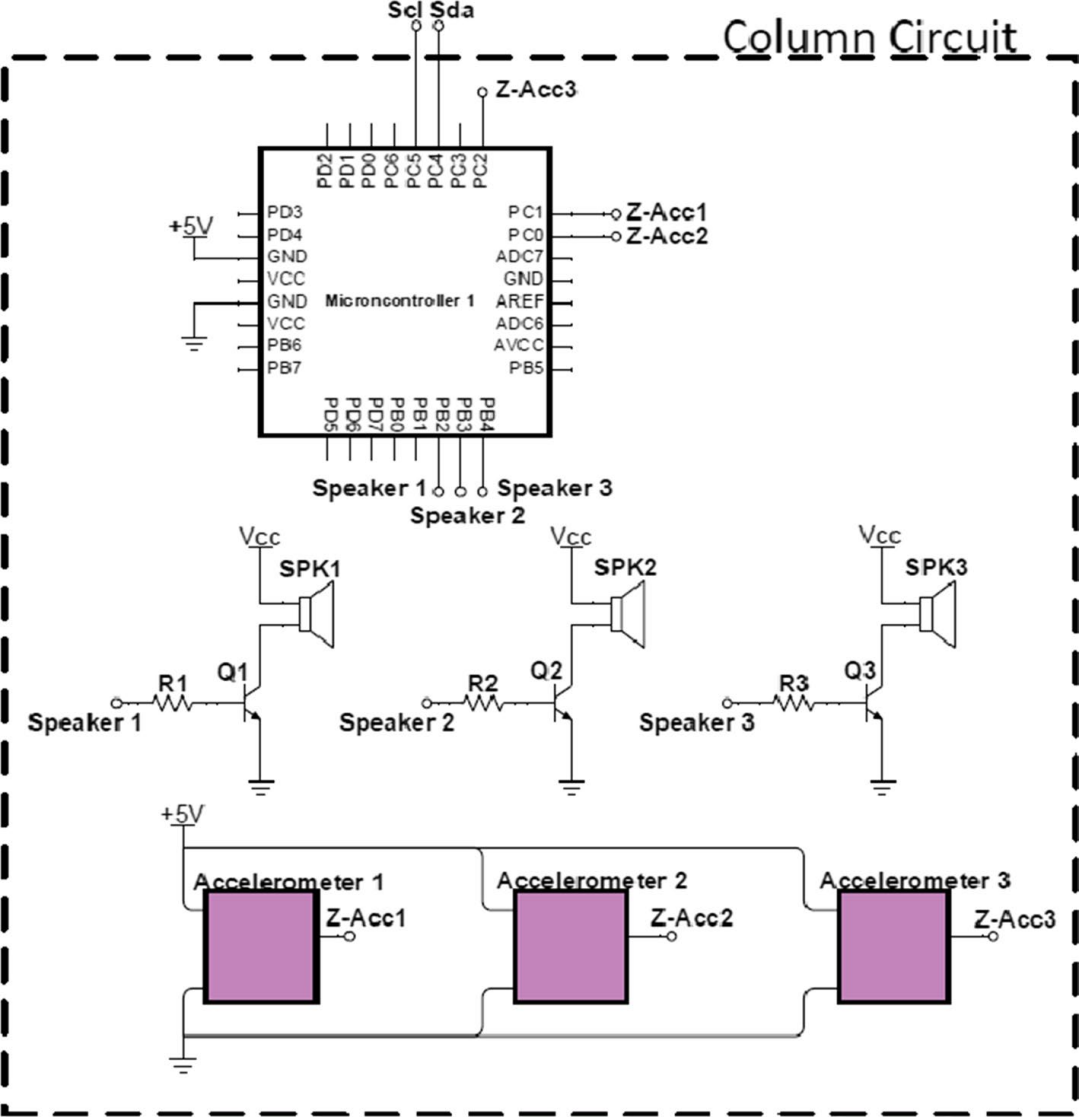
Schematic drawing of one module of four cells from the electronic goal

**Fig. 6:**
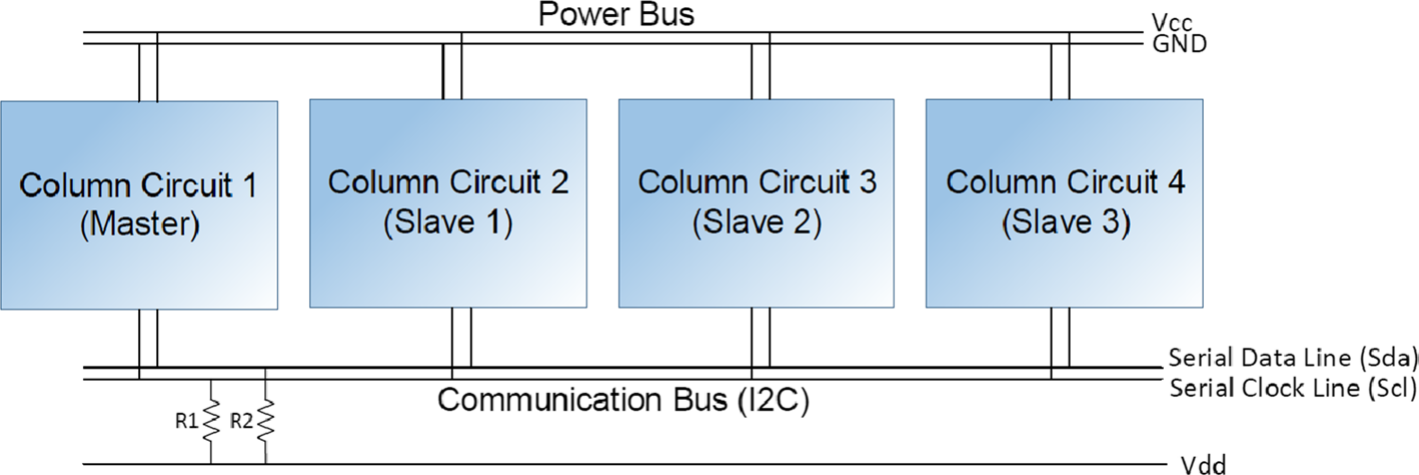
Connection among all modules in the electronic goal

Each column of three cells is treated as a single electronic module as shown in Fig. 5. To detect the impact, an accelerometer is located in the middle of each cell, next to a small 8w speaker. The three accelerometers and the three speakers from a single column are connected to a micro-controller (Arduino UNO) which processes the accelerometer signal from the *z*-axis only (an impact on the cell causes variations on the *z*-axis). Such processing includes a high-pass filter and a threshold to avoid small disturbances that remain after the impact. As shown in Fig. 6, all modules have their own micro-controller, but they are all connected through an $I^2C$ network. The entire system is powered with 5v/7A.

Once the ball impacts the electronic goal, all micro-controllers from each module receive some information from their accelerometers (all cells are joint and therefore transmit movement). To avoid the horizontal or vertical propagation of the vibration through the cells, the *z*-axis of the accelerometers is used to detect and identify the impact of the ball. Each micro-controller calculates the power of the signal from each sensor (based on its amplitude analysis). The cells that experience vibration due to a ball hit emit a sound with volume proportional to the amplitude of the acceleration read from the *z*-axis. Thus, more than one cell could emit a sound depending on the position where the ball impacted. It is to be noted that the electronic goal is designed following the athletes and their trainers’ guidelines and topics such as physiological aspects that might (or might not) be related to the use of the device are not covered in this work.

#### Training modes

**Fig. 7:**
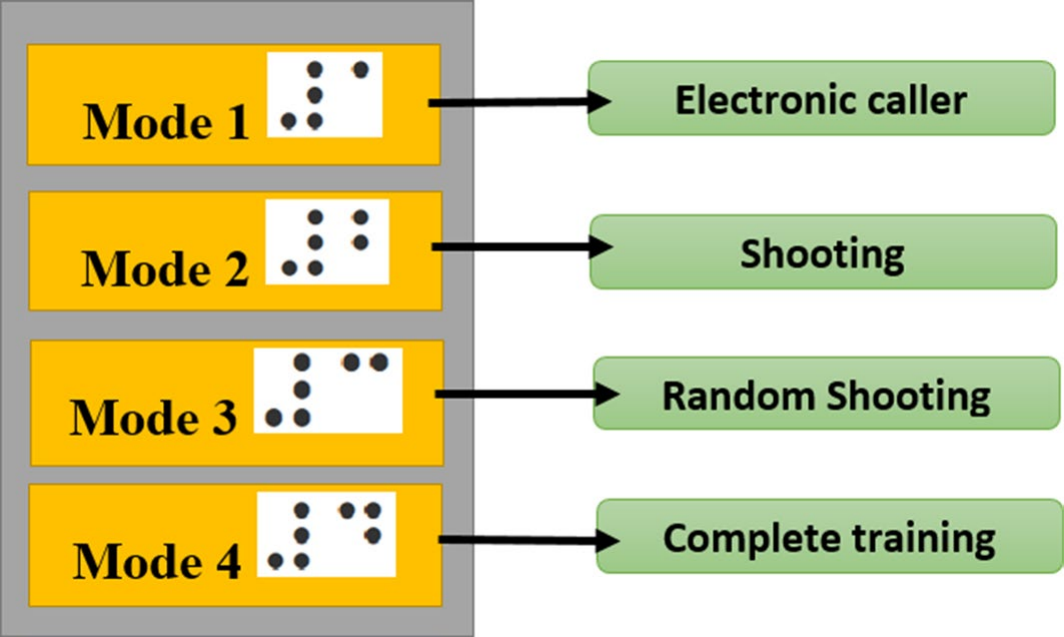
General layout of the training system

Figure 7 shows a general layout of the system. Four training modes were implemented in this work to attend the needs of the athletes. To start, a console with switches serves a human–machine interface between the athlete and the electronic goal. Each switch has in Braille the number of the mode (from 1 to 4), since switch represents one functionality of the system. On a side of the console, a volume knob allows for controlling the volume of all the speakers at the same time. In brief,

- Mode 1: the most intuitive mode is aimed at replacing the *caller*. Each cell from the electronic goal, located in one of the four corners of the grid, emits a unique tone. It performs counter-clockwise with a delay of 1 s between each cell. It starts from the right-down corner cell. Once one cycle (the four corner cells) have been activated, the system waits two more seconds before re-starting the *caller* mode.
- Mode 2: if selected, Mode 1 is first executed (once) for sound localization of the player and the goal. Then, each cell from the goal, starting from the right to left, and from the bottom to up, emits its corresponding sound, with average volume. This mode is repeated until the player switches to modes in the console. Thus, the athlete is able to recognize the sounds from each cell.
- Mode 3: if selected, Mode 1 is again first executed for sound localization as in the previous mode. In this mode, sequentially and from right to left, and from the bottom up, each cell emits its sound and waits 10 s (recommended time by the professional trainers) for a shooting. If the ball impacts on the cell, then the same sound is repeated. Otherwise, the sound of the impacted cell is executed. This mode allows the athlete to memorize the sound associated with each cell and to practice his shooting skills.
- Mode 4: if selected, this mode performs as follows: (i) Mode 1 is executed to sound-localize the athlete according to the goal; (ii) a random cell emits its tone and waits 10 s for a shooting. If no shooting is registered, then the same cell emits its tone a second (and last) time. (iii) Once the player shoots, the sound produced corresponds to the cell where the ball impacted. Since every cell from the grid has its own sound, the player realizes if the ball has impacted in the suggested cell.

The system is designed to add more modes according to the convenience of the players or the recommendations from the professional trainers.

### Questionnaire

To test and validate the proposed electronic goal, seven athletes from the CNFST volunteered to test the goal during practice and penalties shooting at their training field in Santiago, Chile. A questionnaire was presented to each player and consisted of three parts: (i) a consent and some information about the volunteer regarding his impairment; (ii) a pre-questionnaire before using the electronic goal; and (iii) a post-questionnaire after practizing with the electronic goal.

#### Consent and personal details

This part of the questionnaire is mandatory for all volunteers and consisted of the three following items:

i. It was read to all athletes: *By answering this questionnaire I declare that I am aware that the developed system is a tool for my activity and not a prosthetic device and it does not interfere with my body. I also declare that I am aware that the electronic goal is a non-invasive device and it does not represent a risk to my health*. Up to this point, all volunteers answered positively.
ii. *From which type of visual impairment do I suffer?*
iii. *According to IBSA, which type of visual impairment do I suffer?*.

#### Pre-questionnaire

The pre-questionnaire consisted of the four following questions:

i. In a scale 1–5 (being 5 the maximum in importance) identify the need of a *caller* during your training.
ii. In a scale 1–5 (being 5 the maximum in importance) identify your dependence on the *caller*.
iii. Identify your main problem when practicing football soccer.
iv. Identify your main issues when shooting the goal.

The pre-questionnaire served to find the basis for designing the electronic goal and it was fulfilled by the players prior using the electronic goal.

#### Post-questionnaire

After testing the electronic goal in all its functionalities (described in “Training modes” section), the athletes were asked to answer the following points:

i. In a scale 1–5 (being 5 the maximum score) do you feel that the electronic goal has effectively replaced the work of the *caller*?
ii. In a scale 1–5 (being 5 the maximum score) do you feel that the electronic goal gives you more independence when training?
iii. In a scale 1–5 (being 5 the maximum score) did you feel comfortable using the electronic goal for self-training?
iv. In a scale 1–5 (being 5 the maximum score) do you feel that the electronic goal will allow you for a more efficient training?
v. Which one of the four modes do you think is the most important?

Although brief, the questionnaire was aimed at validating the proof of concept proposed in this work.

## Results

**Fig. 8:**
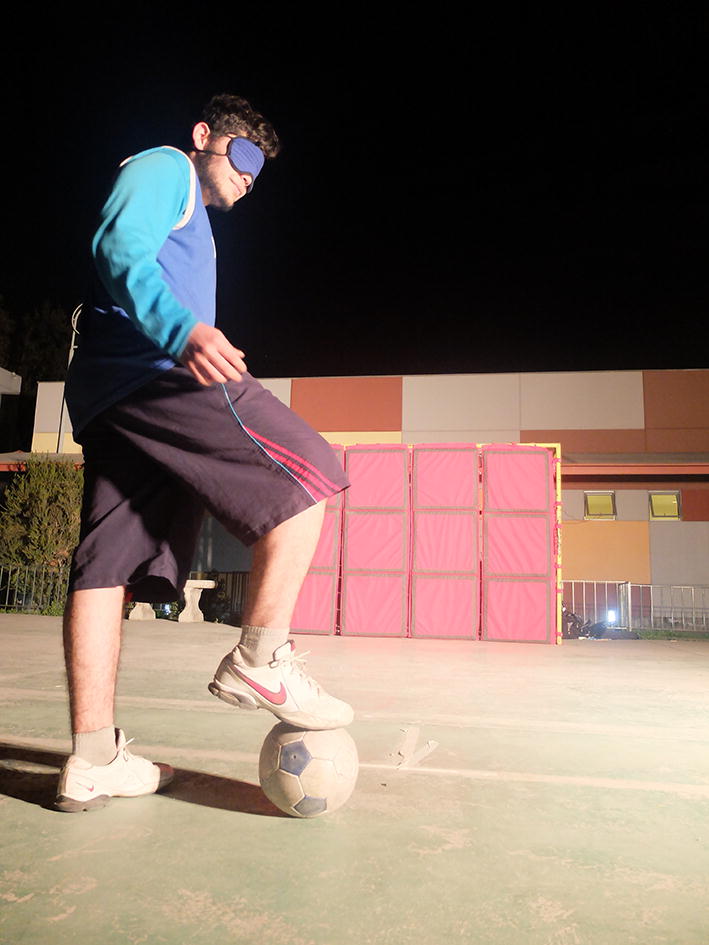
First shooting with the electronic goal

Figure 8 shows one of the athletes in front of the electronic goal. In total 7 athletes from the CNFST volunteered to test the electronic goal in its four modes presented in “Training modes” section. The seven volunteers were asked to answer the questionnaire. The validation procedure was as follows: each athlete was able to shoot the electronic goal up to 25 times from different locations and 25 times from the penalties shooting spot without the assistance of a caller and without the presence of a goalkeeper. Each volunteer experienced the four modes of the system.

### Consent

The seven volunteers freely consented to perform the tests. Their visually impairments vary from damages to retina (at several levels) to tumors. Regarding their category according to the IBSA classification, five athletes were B1 type and two B2 type.

### Pre-questionnaire

**Fig. 9:**
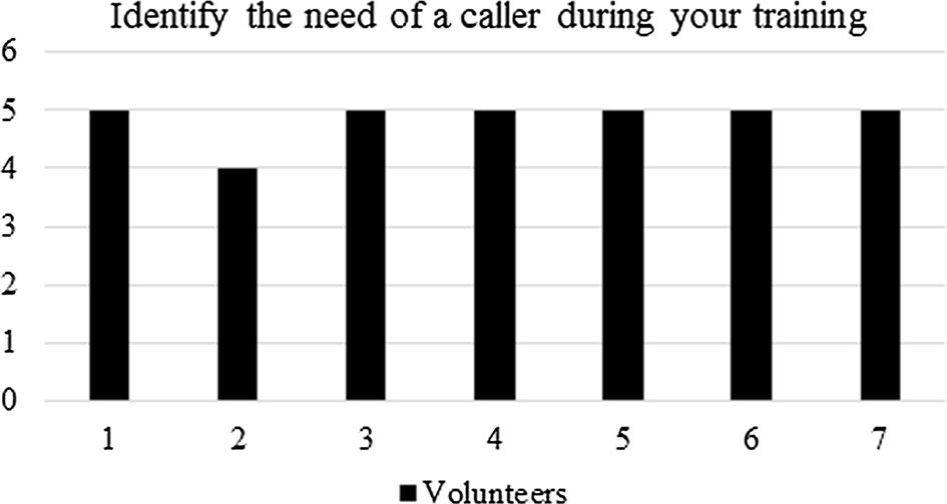
Pre-questionnaire: quantification of the need of a *caller* during training

Regarding the pre-questionnaire, Fig. 9 shows the answers given by the athletes to the evaluation of the need of a *caller* during training. As can be seen, almost all volunteers answered that the *caller* is of maximum need.

**Fig. 10:**
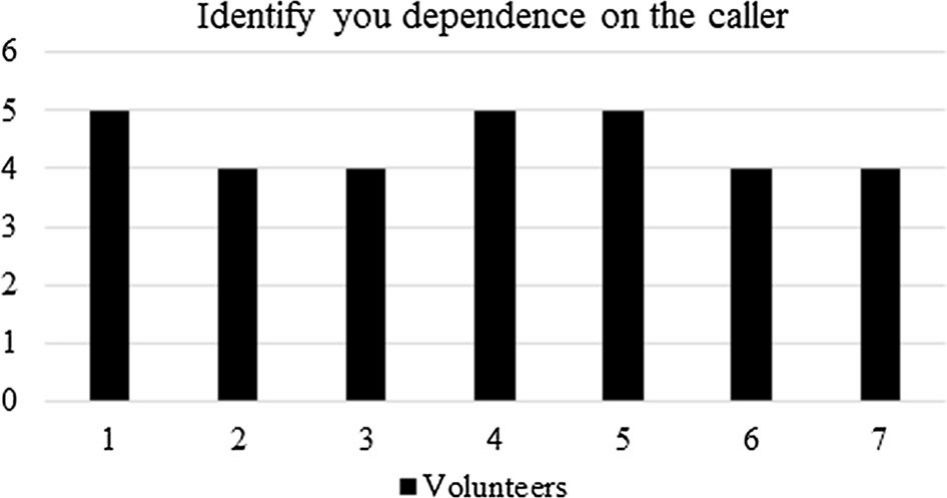
Pre-questionnaire: quantification of the dependence on the *caller*

In Fig. 10, the quantification of the dependence of the athletes on the presence of the *caller* on the field is shown. At first sight, this question resembles the previous one, but it is intended to separate the need of the players regarding the presence of the *caller* during training or a match, and their own dependence (as players) given their skills. As can be seen, answers vary from those shown in Fig. 9. Nevertheless, all answers fall within a score of 4 or 5 (maximum dependence).

Regarding question (iii): identify your main problem when practicing football soccer, the answers varied according to objective and subjective points of view, mainly associated to the athletes’ impairments. Thus, volunteers #1 and #5 answered that their main problem was shooting to the goal from any position; for volunteers #2 and #4, the main problem was orientation within the field; for volunteer #3 was leading the team; for volunteers #5 and #7 was the preparation previous the match or training.

Regarding question (iv): identify your main issues when shooting the goal, volunteers #1, #4 and #5 answered that their main problem is accuracy since they are not able to accurately localize themselves with respect to the goal; and volunteers #2, #3, #6 and #7 answered that their main issue was to pay attention to the *caller*.

### Post-questionnaire

After shooting the goal, the seven volunteers answered the post-questionnaire to measure their improvement when using the electronic goal.

**Fig. 11:**
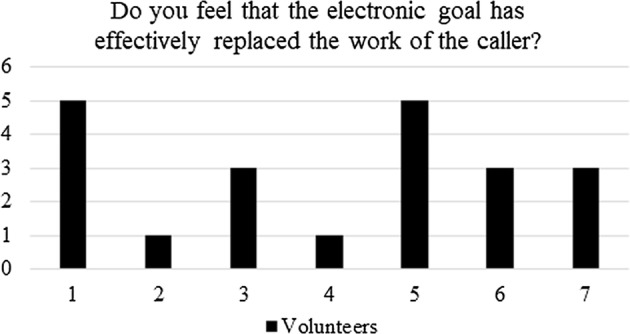
Post-questionnaire: quantification of the replacement of the *caller* during shooting practices

**Fig. 12:**
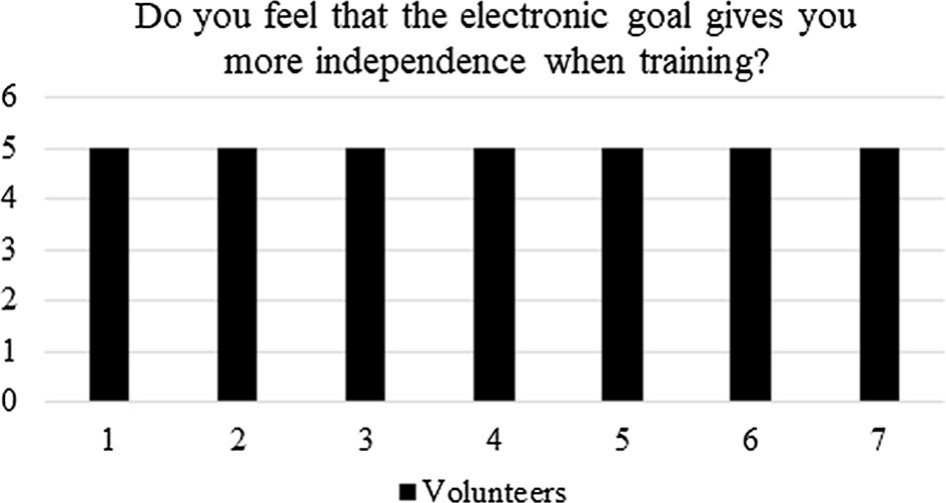
Post-questionnaire: quantification of the self-sufficiency during training

Regarding question (i): do you feel that the electronic goal has effectively replaced the work of the *caller*? Figure 11 shows that in average, the score given by the athletes to this question is 3. The later means that not all the players considered that the electronic goal replaced the role of the *caller*. Although this result can dismiss the hypothesis, Fig. 12 shows the results of the second question: do you feel that the electronic goal gives you more independence when training? As can be seen, all athletes scored the maximum. The latter means that the electronic goal enhances the self-sufficiency of the athletes.

**Fig. 13:**
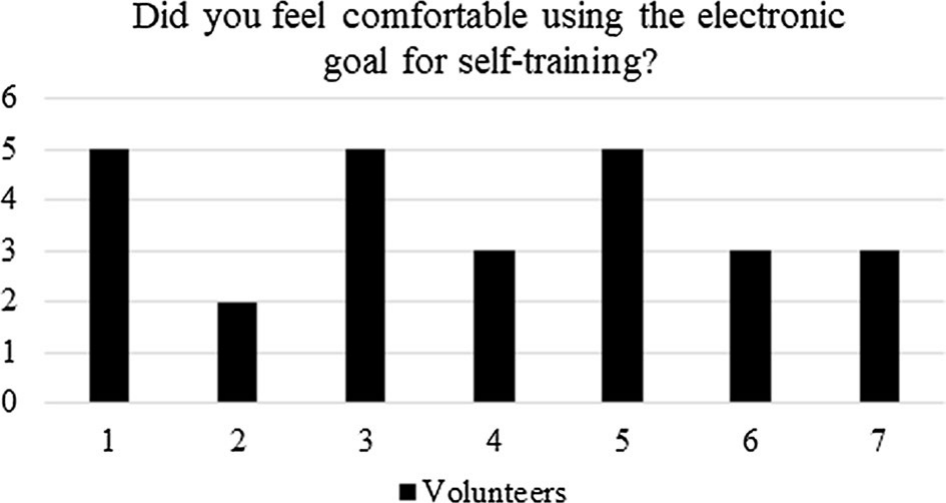
Post-questionnaire: quantification of the comfortability using the electronic goal

Regarding question (iii), associated with the comfortability of using the electronic goal (which implies not only shooting, but also assembling and de-assembling the device), the average score was 3.7 as shown in Fig. 13. Thus, several improvements should be made in order to make the electronic goal more friendly to use, and it could will lead the future work of the authors.

**Fig. 14:**
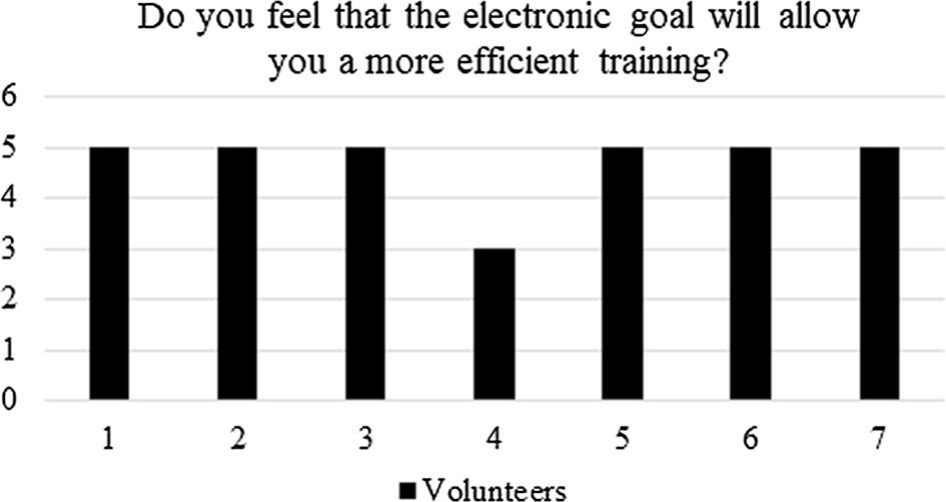
Post-questionnaire: quantification of the efficiency of the electronic goal

In question (iv): do you feel that the electronic goal will allow you for a more efficient training? Most athletes scored the maximum thus validating the concept presented in this work, since the electronic goal is not only to be used on an actual iron goal, but also at home, pending on a wall, and not necessary at the football soccer field. Figure 14 shows the results for question (iv).

**Fig. 15:**
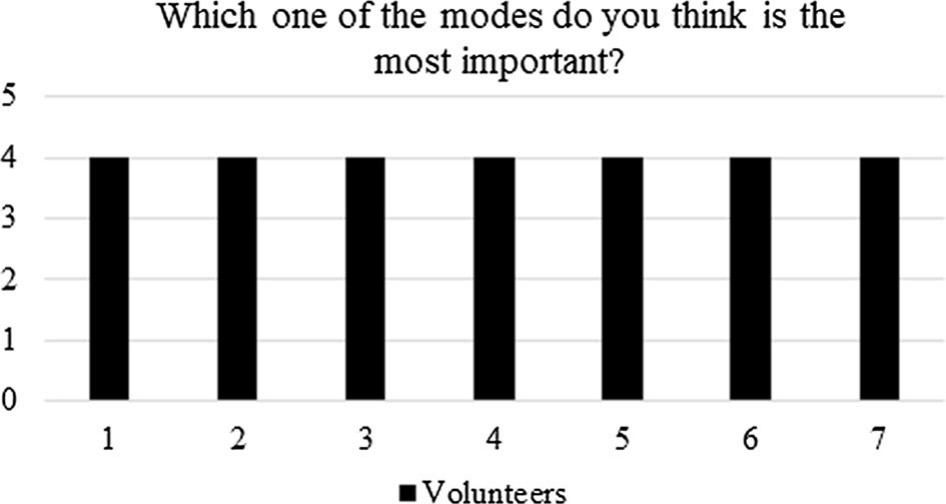
Post-questionnaire: the most important training mode of the electronic goal

Finally, question (v): which one of the four modes do you think is the most important? All athletes agreed that mode 4 is the most convenience for their training (see “Training modes” section), as shown in Fig. 15 and it is expected to be used in their daily training stages.

### Comparison results

To validate the system against a human *caller*, athletes had to shoot 50 times with the electronic goal and 50 times with the human *caller*. Two main results arise:

- With the electronic goal, athletes had a 92% of assertiveness (goals) against 82% with the human *caller*.
- With the electronic goal, athletes had 56% of assertiveness when using Mode 4, against 20% when the human *caller* gave indications where to shoot.

Thus, the electronic goal proved to enhance the accuracy of the shooting training.

## Lessons learned

During the tests, several lessons were learned.

1. Regarding the electronic design, the athletes showed that the fourth mode is the most important and useful one, specifically since it allows them the expected self-sufficiency during training, as evidenced in Fig. 15. The later does not invalidate the other functionalities, but gives a perspective of how to improve, from a hardware point of view, the electronic goal—i.e., by avoiding the other modes.
2. From a teamwork perspective, one of the main obstacles faced was the fact that the *caller* is part of the team and athletes at first felt that the electronic goal was trying to replace the *caller*. After using the device, as can be seen in Figs. 13 and 14, the players realized that the system aim was to enhance the self-training.
3. From a tuning perspective, during the first round of tests, all sounds from the grid were programmed with a frequency higher than 1.5 kHz. However, the athletes asked to lower such frequencies. After tuning, all sounds remained within a wideband of 300 Hz, between 800 Hz and 1.1 kHz, agreed by the athletes.
4. The comfortability of using the electronic goal is shown in Fig. 13. Clearly, the answers given by the players were not conclusive. However, this result is associated with the assembling and de-assembling process performed by the athletes. Although the processes were successfully performed by the volunteers, they pointed out that the electronic goal was too heavy. Each column weight was about 5 kg. Therefore, the electric goal should be built with lighter materials if portability is pursued.
5. In general, the electronic goal fulfilled its purpose. However, a long test trial is still needed to evaluate the effects of the electronic goal on the evidenced abilities of the players.

## Conclusion

In this work, an electronic goal for visually impaired football soccer athletes was presented and tested. The goal followed a modular grid-based design to enhance the self-sufficiency of the players and it was tested with the CNFST-Chile. The aim of the goal was to overcome the need of a *caller*, an extra assistant located behind the iron goal who is in charge of generating sounds to allow the players to locate with respect to the goal, via the use of sound. The electronic goal had four functionalities: the replacement of the *caller* and three modalities for self-training, in which each cell from the gridded electronic goal emitted a characteristic sound both as a way to send references to the player as well as a feedback indicating where the ball hit the goal.

According to the experimental results and the questionnaires performed to have a measure of the impact of the device, the athletes found the system convenient and useful, especially for practizing shootings since it enhanced their assertiveness up to 92%. In the presence of a *caller*, the accuracy was 82% for 50 shootings. The population was formed by 7 volunteers who agreed about the comfortability of using an electronic goal (all of them agreed that they felt enhanced their self-sufficiency). Although the electronic goal had previously programmed sounds, most players asked to set their own convenient sounds. Finally, among the four modes implemented in the electronic goal, the fourth mode (directly related to shooting activities) was the one that the athletes considered as the most relevant. The latter can further enhance the design of a portable goal.

## Data Availability

Data sharing is not applicable to this article as no datasets were generated or analyzed during the current study.
